# Solvent Extraction as a Method of Recovery and Separation of Platinum Group Metals

**DOI:** 10.3390/ma16134681

**Published:** 2023-06-28

**Authors:** Karolina Pianowska, Joanna Kluczka, Grzegorz Benke, Karolina Goc, Joanna Malarz, Michał Ochmański, Katarzyna Leszczyńska-Sejda

**Affiliations:** 1 Łukasiewicz Research Network-Institute of Non-Ferrous Metals, Sowińskiego 5, 44-100 Gliwice, Poland; grzegorz.benke@imn.lukasiewicz.gov.pl (G.B.); karolina.goc@imn.lukasiewicz.gov.pl (K.G.); joanna.malarz@imn.lukasiewicz.gov.pl (J.M.); michal.ochmanski@imn.lukasiewicz.gov.pl (M.O.); katarzyna.leszczynska-sejda@imn.lukasiewicz.gov.pl (K.L.-S.); 2 Department of Inorganic Chemistry, Analytical Chemistry and Electrochemistry, Faculty of Chemistry, Silesian University of Technology, B. Krzywoustego 6, 44-100 Gliwice, Poland; joanna.kluczka@polsl.pl

**Keywords:** platinum group metals, solvent extraction, noble metal recovery

## Abstract

Platinum group metals (PGMs) are a group of six metals with high market value and key importance to many industrial sectors. Due to their low prevalence in the Earth’s crust and high demand, these metals have been recognized as critical materials for many years. Along with economic development, the natural resources of the platinum group metals are gradually depleting, which is accompanied by the need to recover PGMs from secondary sources. The solutions resulting from the processing of such materials are characterized by high content of impurities and low content of precious metals. For this reason, in order to obtain pure metals, it is extremely important to choose an effective, selective method for the recovery and separation of the platinum group metals. This review focuses on the most important aspects of the characteristics of the PGMs, including their properties and occurrence, the processing of natural and secondary raw materials and the role of liquid–liquid extraction in the selective separation of metals from this group, not only on a laboratory scale but, above all, on an industrial scale. In addition, this study collects information on the most commonly used, commercially available extractants, based on current reports, taken from the scientific literature.

## 1. Introduction

**Figure 1:**
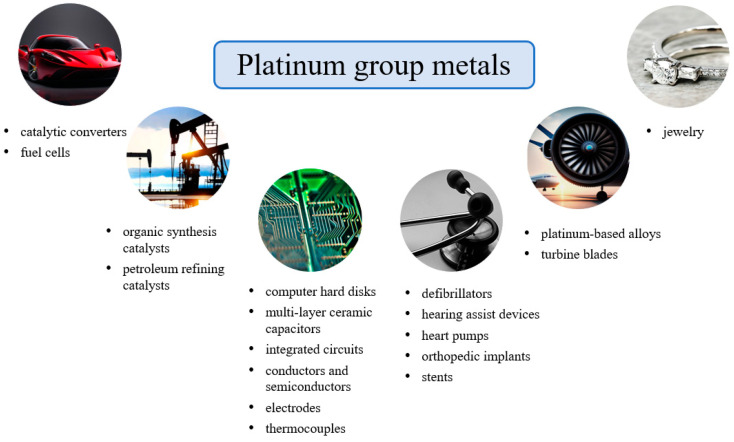
The applications of platinum group metals. (Source: own elaboration based on literature data [1,2,3,4,5,6,7,8,10,11,12]).

Platinum group metals (PGMs) are a group of metals with high market value and low prevalence in the Earth’s crust. Physically, they are grayish-white metals (except for osmium) with relatively high hardness and brittleness. Platinum group metals are most popular due to their unique physical and chemical properties, such as high chemical and thermal resistance, very good thermal and electrical conductivity and resistance to corrosion and oxidation. Thanks to their unique aforementioned properties, PGMs have found application in many industrial sectors, such as the automotive, chemical, petroleum, electrical and electronics, jewelry, aerospace, medical, dental and pharmaceutical industries (Figure 1) [1,2,3,4].

PGMs play a particularly large role in catalysis. For many years, the largest consumer has been the automotive industry, which uses them to produce automotive catalysts that prevent harmful emissions of carbon oxides, nitrogen oxides and hydrocarbons [2,5,6]. In the chemical and petroleum industries, they have found use in processes such as reduction, reforming, hydrogenation, isomerization and conversion [1,2,3,7]. Despite the many applications and high market demand, access to PGM natural resources is limited, due to the low occurrence of these metals in the Earth’s crust. This in turn also affects the high price of these metals [2,5,8]. According to reports from Johnson Matthey, prices for PGMs in January 2023 reached $1107.00/oz for Pt; $1855/oz for Pd $12,400.00/oz for Rh; $4800.00/oz for Ir; and $475.00/oz for Ru [9].

## 2. The Main Sources of PGMs

**Figure 2:**
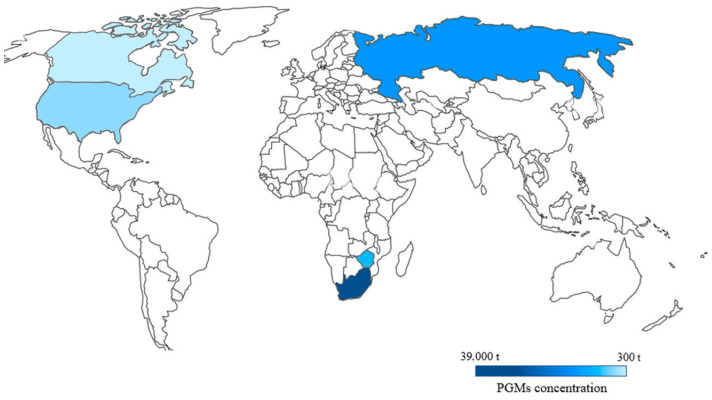
The locations of the platinum group metals’ main resources by country. (Source: own elaboration based on literature data [15]).

As mentioned earlier, PGMs are elements with low prevalence in the Earth’s crust, so they belong to the critical metals. Currently, the main supplier of PGMs is South Africa, where most of the world’s reserves are located, estimated at approximately 63,000 tons in 2020. Other locations with rich deposits of these metals include Russia (5500 tons), Zimbabwe (1200 tons), the United States (900 tons) and Canada (310 tons). In other parts of the world, including Scandinavian countries and East Asian countries, natural resources of platinum metals are also present, but data on the exact amounts of PGMs given in metric tons are rarely available [13,14]. Figure 2 shows the countries with the largest natural resources of PGMs [1,2,11,15].

The form of a platinum group metal in natural deposits largely depends on the geographical location. Typically, PGMs accompany sulfide and arsenic ores of copper and nickel. The world’s largest deposits—the Merensky Reef located in the Bushveld Complex—are magmatic sulfide deposits of Ni-Cu-PGM. PGMs in their native form are found mainly in South Africa, the Ural Mountains and Colombia [1,2,5,16].

Depending on the type of ore, the PGM concentrations in the deposits can exhibit different values—the richest South African ores contain less than 10 g of PGMs per ton of ore. Such low content of precious metals, even in the case of the richest deposits, necessitates an enrichment process involving a series of physical, pyro- and hydrometallurgical processes. The first stage of processing involves the extraction of ores by conventional underground or open pit techniques. The material is then processed by crushing and grinding. Enrichment of the material is usually carried out using flotation, gravity separation and pyrometallurgical methods, i.e., smelting and conversion. The next stage is hydrometallurgical pretreatment, during which contaminants such as Fe, Co, Ni and Cu are removed. As a result, a rich PGM concentrate is obtained, which is destined for further refining and the recovery of individual PGMs [2,3,5,8,16,17].

Since continuous economic development causes an increase in the demand for precious metals, the natural sources are gradually depleting. Therefore, it is necessary to search for new means of obtaining them. The possibility of recovering precious metals by processing secondary sources, such as used automotive catalytic converters and industrial catalysts, electronic and computer components or aircraft turbines, is becoming a particularly important issue. Among those mentioned, used automotive catalytic converters and industrial catalysts are a particularly valuable source of PGMs. For this reason, the recycling of such materials has been the focus of researchers’ attention in recent years. Moreover, a number of corporations—including Umicore, BASF, Johnson Matthey and Nippon PGMs—have already developed commercially used processes for the recovery of PGMs from secondary sources. Similar to the processing of primary sources, the recycling of waste materials usually uses both pyrometallurgical and hydrometallurgical processes. The sequence of individual process operations and process conditions is adapted to the chemical composition and type of raw material used. However, most of the processes described in the scientific literature concern the recycling of spent catalysts [5,11,12,18].

The first stage of processing usually involves the crushing, grinding and portioning of the feedstock, followed by melting in the presence of a flux, reductant and collector (usually Cu, Ni, Pb or Fe) or, in the case of the hydrometallurgical method, pre-leaching. The pretreatment is aimed not only at concentrating the PGMs but also at removing residual organic matter from the catalytic processes or elements of the support and matrix that limit the leaching efficiency of the PGMs [5,11,12,18].

**Figure 3:**
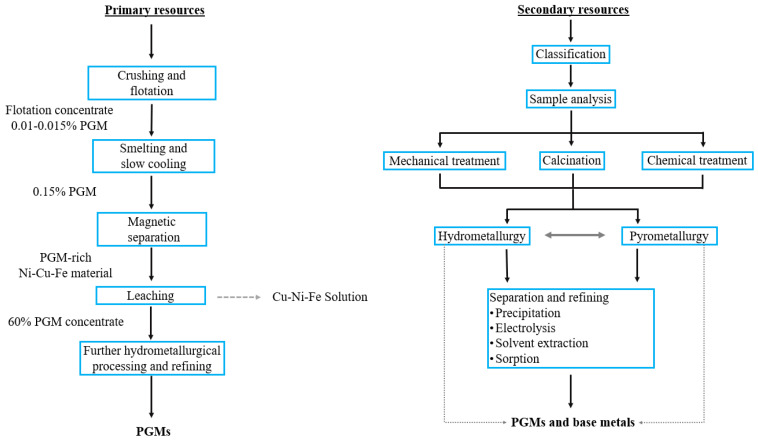
The technological schemes of processing primary and secondary materials. (Source: compiled from [3,18]).

The following Figure 3 shows a comparison of the general technological schemes for the processing of primary and secondary materials.

## 3. Industrial Methods for the Platinum Group Metals’ Separation

The processing of precious metal sources usually leads to solutions characterized by low precious metal content and high content of other base metals. In addition, the strong similarity of platinum group elements’ properties and their high chemical resistance still make the selective separation of precious metals one of the greatest challenges in chemical technology. Commonly used pyrometallurgical processes are only applicable at the pretreatment stage to produce concentrates. Unlike hydrometallurgical methods, they do not lead to pure and separated platinum group metals. Furthermore, pyrometallurgical methods suffer from a number of disadvantages, including high energy consumption or the generation of large quantities of volatile and toxic combustion products. Hydrometallurgical methods do not require such high temperatures and provide relatively low costs, both on a small and large scale, while maintaining high efficiency and easy process control. In addition, the liquid waste produced by hydrometallurgical methods can be purified and returned to different processes [5,12].

Therefore, PGMs are a group of metals that can only be obtained in their pure state by hydrometallurgical methods such as precipitation, solvent extraction or ion exchange. Of the aforementioned, the oldest are the precipitation methods, which were used in the world’s refineries until the mid-1970s. The separation of the individual precious metals was carried out by means of a series of precipitation reactions. However, the large number of impurities and interfering co-precipitation reactions necessitated the use of multiple refining operations, making the process very tedious and time-consuming [3,7].

In later years, solvent extraction became particularly important. The advantages of extraction methods, such as high reaction rates, high selectivity and relatively low reagent prices, have resulted in this technique being successfully used in precious metal refineries worldwide, including the Anglo Platinum Corporation, the Rand Refinery in South Africa and Johnson Matthey in England. In addition, a number of effective PGM extractants are known, such as primary, secondary and tertiary alkyl amines, alkyl sulfoxides, organophosphorus compounds (both chelating and solvating) or pyridine derivatives [3,7,19,20,21,22,23].

Most available process schemes involve leaching the feedstock materials in concentrated hydrochloric acid with the addition of chlorine gas as an oxidant. The first separation step is usually the solvent extraction of gold, followed by palladium or platinum. The remaining PGMs are recovered at the beginning or end of the entire process. In each case, the separation leads to pure solutions of the relevant metals, which are reduced to a pure powder or sponge. The final products are usually obtained by remelting to produce ingots, pellets or rods [1,5,6]. In the literature reports, solvent extraction as a method to recover precious metals was first used by the International Nickel Company (INCO) in the UK, where dibutyl carbitol was used as the extraction agent for the separation of gold from chloride solutions [7,24].

Another example is the Anglo Platinum refinery in South Africa, which recovers gold using methylisobutylketone. In addition, South Africa has developed an extractive gold refining process—the Minataur™ Process—for the processing of raw materials with Au content of 50–99%. This process is used in South Africa, Algeria and Dubai [7].

Palladium extraction usually proceeds using organosulfides as extractants, which selectively separate Pd from other noble metals. The exception is gold, which can be co-extracted with palladium; therefore, Au extraction is usually the first step in a flowchart. The Pd extraction process is characterized by very high efficiency—usually less than 1 mg/L of Pd remains in the raffinate [3,5,7].

The recovery of Pd from extracts is carried out using ammonia, from which Pd(NH_3_)_2_Cl_2_ is precipitated after acidification with HCl. The final step leading to Pd in its metallic form is usually calcination. Reduction can also be carried out using HCOONa or HCOOH. Literature data indicate that the global refiners, i.e., INCO and Degussa, use dioctyl and dihexyl sulfides for Pd extraction. In the case of Johnson Matthey and Anglo Platinum, β-hydroxyoxime with the addition of amines as phase transfer catalysts is used [3,5,7,8].

The most commonly used extractants for platinum are tributyl phosphate and amine compounds. At the Johnson Matthey refinery, trioctylamine is used to extract Pt from gold- and palladium-depleted solutions. During Pt extraction, it is extremely important to control the reduction potential due to the possibility of co-extraction of Ir(IV). For this reason, the Pt extraction step is usually preceded by the reduction of Ir(IV) to Ir(III) with the use of sulfur dioxide [7,24].

An alternative method to solvent extraction is sorption of the PGMs on solid supports. Sorption, similarly to solvent extraction, is characterized by high efficiency and selectivity, especially in the case of very dilute solutions with low content of precious metals, as well as the possibility of bed regeneration and a long lifespan of the resins, which is an important economic factor [3,18,25].

In practice, in world refineries, both methods are used interchangeably, depending on the composition of the starting solution, and they have been a permanent element of technological separation schemes for years. The most popular and effective noble metal sorbents include ion exchange resins such as AMBERSEP™ 43600, LEWATIT^®^ K 6362, LEWATIT MonoPlus TP 214 or Puromet MTS9100. A slightly different type of sorption, called molecular recognition technology (MRT), is characterized by products from the SuperLig series, widely used in global refineries around the world. For Pd sorption, a product with the trade name SuperLig 2 is used; for Pt, SuperLig 133 is used; for Ir, SuperLig 182 is used, and for Rh, SuperLig 190 is used [3,18,25].

**Figure 4:**
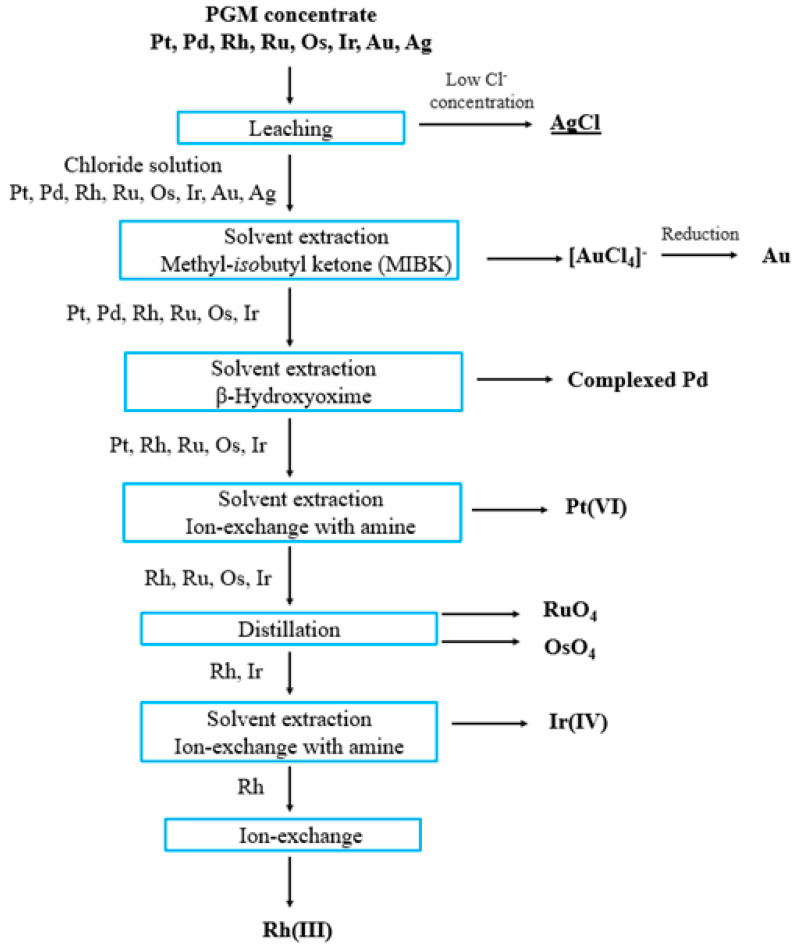
The technological schemes of the separation of PGMs. (Source: compiled from [3,24]).

An example of a technological diagram of PGM recovery and separation is presented below (Figure 4).

## 4. Solvent Extraction

Solvent extraction, also known as liquid–liquid extraction, is an effective method for the separation of metals from multi-component solutions. The technique involves the transfer of components from one phase, usually aqueous, to a second phase, an organic one, which is immiscible with the first phase. Once equilibrium is reached, the two phases are separated, resulting in an extract and a raffinate. The loaded organic phase (extract) then undergoes stripping to recover the extracted component, and further regeneration. The metals contained in the re-extraction solution can then be reduced to transform them into the metallic form or a specific chemical compound. The purified organic phase can be directed for reuse in a subsequent extraction process. As the hydrometallurgical treatment of natural or secondary sources of precious metals produces acidic chloride solutions, the mechanism of PGM solvent extraction is closely linked to the chemistry of chlorocomplexes [7,26].

### 4.1. Platinum Group Metals in Chloride Solutions

Hydrochloric acid is the most commonly used and economically viable leaching agent; moreover, the chemistry of the platinum group metals’ chlorocomplexes is very well understood, which is crucial for downstream separation and refining steps.

As precious metals form a wide range of chlorocomplexes (Table 1), the most important factors responsible for their appearance and durability are the pH, redox potential, chloride concentration and the so-called solution ageing effect, which is responsible for the formation of aquacomplexes in low-acid solutions with low chloride concentrations. Studies indicate that the type of PGM chlorocomplex play an important role in the processes of selective extraction or ion exchange, which is related to the different geometry and charge density and, consequently, the different reactivity and kinetics of the processes occurring in aqueous solutions. For example, in the case of processes occurring according to the ion exchange mechanism, the general tendency for the formation of ion pairs with extractant molecules follows the series [MCl_6_]^2−^ > [MCl_4_]^2−^ >> [MCl_6_]^3−^ >> aquacomplexes. This effect is mainly due to the differences in the charge density of different forms of chlorocomplexes and therefore their tendency to hydrate. Chlorocomplexes with a high charge density more easily attract water molecules, forming a thick hydration shell, which in turn negatively affects the coulombic attraction and the formation of ion pairs with the corresponding counter ions. Furthermore, studies also indicate that low temperatures and long storage times of solutions favor the formation of kinetically inert, hydrated forms of chlorocomplexes [3,26,27].

### 4.2. Solvent Extraction Mechanisms

As mentioned earlier, solvent extraction is an effective method for the separation of PGMs based on their chlorocomplex chemistry. As a result of the contact between the two phases, PGMs migrate from the aqueous phase to the organic phase, which is usually a synergistic effect of electrostatic attraction and molecular processes. The extraction process can follow three main mechanisms:

- solvation—where water molecules surrounding a metal are replaced by molecules of the extractant;
- anion exchange—involving the formation of electrically neutral ion pairs between the anions of chlorocomplex MCl_x_^n−^ and the positively charged, basic molecules of the organic compound;
- compound formation—a Pd-specific mechanism [27].

Standard molecular extractants, depending on the type of compound, can extract platinum from aqueous solutions by any of the above-mentioned mechanisms. Ammonium- or phosphate-based extractants usually act by either an anion exchange or solvation mechanism [18,27]. 

In the case of ionic liquids, extraction can take place according to the ion exchange mechanism, where the anionic group of the ionic liquid is replaced by the chlorocomplex anion, but also through platinum coordination, association or other reactions based on the exchange of individual ions. In practice, in the case of aqueous liquid ionic phase systems, the extraction reactions have a more complicated course than in the case of classical extractants. Reactions explaining the course of each of the discussed mechanisms are shown in Table 2 [3,18,27,28].

### 4.3. Popular Agents for the Extraction of Platinum Group Metals

Among the commercially available, effective PGM extractants, four main groups of extractants can be distinguished:

- organophosphorus extractants;
- amine-based extractants;
- sulfur-based extractants;
- oxime extractants.

The most popular phosphorus-based extractants include, among others, the already mentioned tributyl phosphate or trioctylphosphine oxides with branched or linear alkyl chain structures, widely known under the trade names Cyanex 921 and Cyanex 925. Tributyl phosphate is also used as a modifier in the extraction process, where it facilitates phase separation in mixed systems and prevents the formation of a third phase [17,18,25,29].

The second largest group of extractants is amine extractants, which, due to their structure, can be divided into primary, secondary and tertiary amines and quaternary ammonium salts. One of the most commonly used extractants is trioctylamine (Alamine 336, Alamine 300) and its derivatives [3,7,19,27,28].

Sulfur extractants, such as dioctyl sulfide, find application in the selective extraction of Pd. Moreover, they are characterized by high stability in strongly acidic environments [26,27,28].

Another well-studied and commercially available group of compounds is the oxime extractants, known under the trade names of the LIX series. These types of extractants are particularly effective in the selective extraction of Pd, where α-hydroxyoxime (LIX 63) and β-hydroxyoximes (LIX 860, LIX 84I, LIX 65N, etc.) are the most widely used [30,31].

With the deepening trend towards environmental protection, recent years have seen the increasing use of ionic liquids such as Cyphos IL 101 and Aliquat 336 in the extraction of the platinum group metals from aqueous solutions. Ionic liquids are categorized as so-called ‘green solvents’ due to their properties, such as low volatility, non-flammability or regenerability. Furthermore, due to their ability to dissolve many substances, they have also found application in organic synthesis, drug production, catalytic reactions, liquid chromatography or electrochemistry. From a chemical point of view, ionic liquids are molten salts that remain liquid at temperatures below 100 °C and are usually composed of large organic cations and inorganic anions. Due to their increasing popularity, ionic liquids are characterized by high market availability and diversity of structures. Due to the structure of the cationic part, we can distinguish between ammonium, imidazole, phosphonium, pyridine, piperidine, pyrrolidine, betaine and guanidine liquids, among others. The anionic part usually consists of simple inorganic groups, i.e., SO_4_^2−^, Cl^−^, Br^−^, I^−^, NO_3_^−^ or fluoroanions such as PF_6_^−^, BF_4_^−^, F^−^, NTf₂^−^ (bis(trifluoromethanesulfonyl)imide). Since classical ionic liquids do not always show selectivity towards specific PGM complexes, in recent years, attempts have been made to use anions containing P, N, S and O atoms or their combinations to increase the selectivity. Most research on the use of ionic liquids for the liquid extraction of platinum group metals focuses on the use of their dilute organic solutions, in which the role of the solvent is played by popular non-polar or semi-polar aliphatic and/or aromatic hydrocarbons. Due to their unique physicochemical properties, the addition of ionic liquids to classical organic solvents creates an ideal environment for the selective separation of precious metals. The charged particles of ionic liquids provide stronger interactions between the active ligands contained in the organic phase and the PGM molecules present in the aqueous solution, consequently increasing the efficiency of the separation of platinum group chlorocomplexes. In the past, studies on the use of undiluted ionic liquids were also undertaken, but due to the high viscosity of these substances and difficult mixing, resulting in slow mass transfer, the use of this type of system, especially in industrial conditions, is limited [19,20,27,28,32,33,34,35,36].

One of the less frequently mentioned, but equally important, advantages resulting from the use of ionic liquids in extraction systems is their surface-active effect, which allows one to reduce the surface tension and increase the contact between the phases [27].

**Figure 5:**
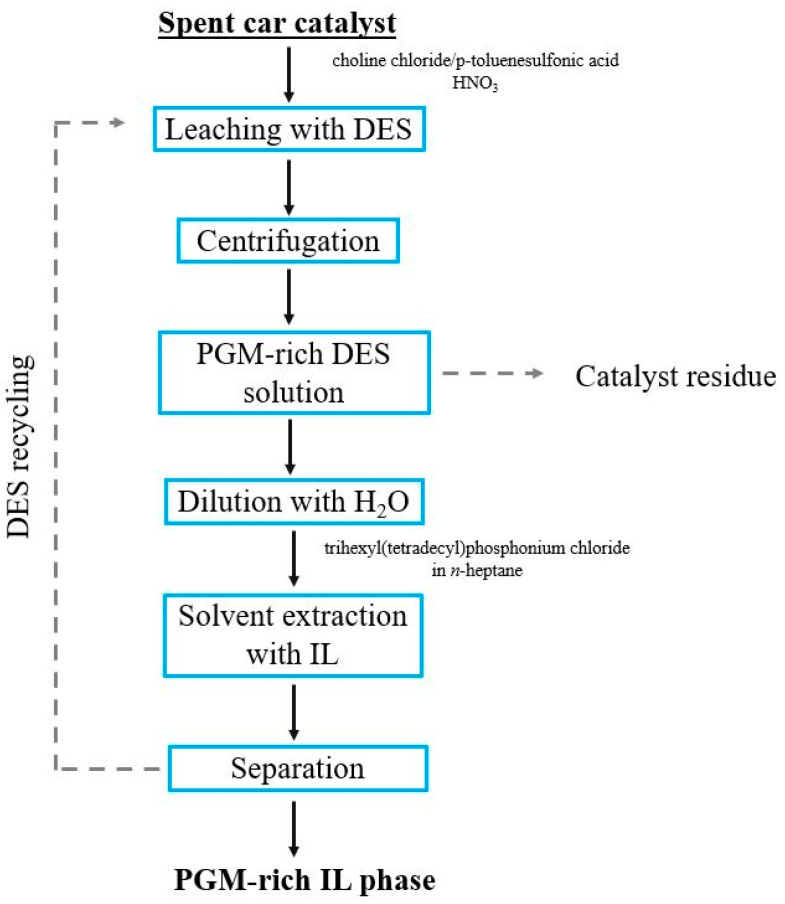
Scheme of PGM leaching and solvent extraction process with DES and IL. (Source: compiled from [41]).

Another group of compounds that has aroused great interest among researchers in recent years is deep eutectic solvents (DES), which are characterized by similar properties to ionic liquids, while, at the same time, being often easier and cheaper to obtain. Due to the low toxicity and often biodegradability of this class of chemical compounds, deep eutectic solvents, similar to ionic liquids, are considered green solvents. Chemically, DES are mixtures consisting of two types of compounds, one of which acts as a hydrogen bond donor (HBD) and the other as a hydrogen bond acceptor (HBA). The presence of hydrogen bonds, in turn, leads to a decrease in the melting point, thanks to which these mixtures remain liquid at room temperature and can be used as leaching or extracting agents in the case of highly eutectic hydrophobic solvents. The studies of PGM solvent extraction with the use of DES conducted in recent years included systems containing, as acceptor factors, e.g., menthol, trioctylphosphine oxide, choline chloride, tetraoctylphosphonium bromide, tetraoctylammonium bromide, tetrabutylammonium bromide, and, as donors, terpenes and saturated alcohols, as well as organic acids including hexanoic, heptanoic, octanoic and nonanoic acids [37,38,39]. For example, research published in 2021 indicated that deep eutectic solvents based on a quaternary ammonium salt, such as methyltrioctylammonium chloride, and saturated fatty acids or saturated fatty alcohols (hydrogen bond donors) are effective extractants for palladium from chloride solutions [40]. In another study, published in the same year, scientists proposed a methodology for the recovery of Pt, Pd and Rh from spent catalysts (Figure 5), in which a deep eutectic liquid—choline chloride/p-toluenesulfonic acid with HNO_3_ addition—was used as the leaching agent, and an ionic liquid, i.e., trihexyl(tetradecyl)phosphonium chloride, as a PGM extractant from a deep eutectic solution [41].

As indicated above, there are many effective classes of compounds for use in the solvent extraction of the platinum group metals. Since solvent extraction is a popular technique for the separation of individual elements from aqueous solutions, most extractants are readily available on the market. Among the main suppliers of extractants, the following companies can be distinguished: TCI Chemicals, abcr GmbH, Thermo Scientific, Sigma-Aldrich, Biosynth, Solvay, Stream, Acros Organics and others.

The table below presents a list of the most commonly used organic extractants, including ionic liquids, used in the separation of metals from the platinum group (Table 3).

**Figure 6:**
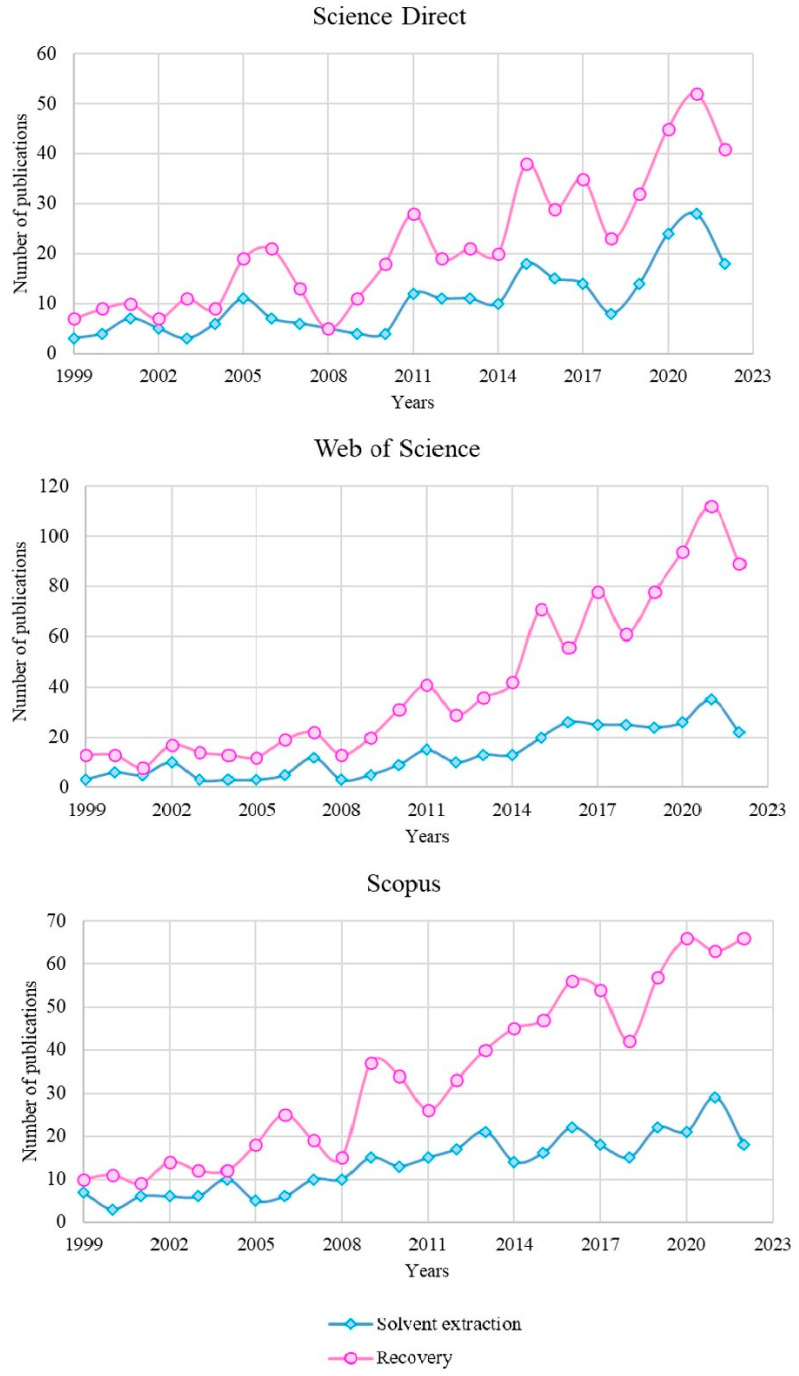
The frequency of publication of articles on solvent extraction and PGM recovery in 1999–2022 by database.

The data presented in Table 3 indicate that there are many effective and commercially available platinum group extractants. Despite the high popularity of extraction techniques and their many years of history in the field of the selective separation of platinum group metals, this technique still arouses widespread interest, as evidenced by the constantly incoming, new scientific publications. Furthermore, from year to year, there is a significant increase in published research in the field of the recovery of these metals. Figure 6 presents data from the years 1999–2023, collected on the basis of search results for selected phrases in the ScienceDirect, Scopus and Web of Science scientific databases. In order to further narrow the range of obtained results, the most important keywords were indicated, such as “solvent extraction”, “recovery” and “platinum group metals”. The received data indicated that the total number of published works on the extraction of the platinum group metals in 2021 and 2022 amounted to 47–57 articles, depending on the search engine and the entered phrase. For comparison, the average number of publications in 1999–2010 was approximately 6 per year.

## 5. Conclusions

The analyzed literature data clearly indicate that solvent extraction is a very effective and selective method for the separation of the platinum group metals from multi-component solutions, thanks to which this technique has been a permanent element of the technological schemes of precious metal refineries around the world for years. The key aspect is not only the high selectivity of the process but also the purity of the metals and the compounds obtained, the relatively short refining time, the possibility for the recycling and regeneration of extractants and the small amount of waste generated. 

The analysis of search results displayed in popular scientific databases indicates that, in recent years, the interest in PGM solvent extraction has increased significantly, with most of the undertaken extraction research currently focusing on the use of ionic liquids and deep eutectic liquids for the effective recovery of platinum group metals from aqueous solutions.

Thanks to the well-known mechanisms of the extraction process and the chemistry of chlorocomplexes, it is possible to design and synthesize new, increasingly effective classes of compounds that can be used in the selective recovery of these valuable metals. Most of the available scientific research, however, focuses on the separation of these metals from synthetic solutions, which, due to their simple composition and lack of other impurities, are not able to fully reproduce the conditions prevailing in technological solutions, which are multi-component systems, characterized by high concentrations of other base metals. Furthermore, continuous economic development and stricter restrictions in the field of environmental protection highlight the need to recover platinum group metals from secondary sources, where used car and industrial catalysts are extremely valuable materials. The recycling of this type of waste allows the recovery of the platinum group metals with relatively low energy expenditure and a small amount of material needed, where, in the case of natural sources, in order to obtain only a few grams of PGM, it is often necessary to process several tons of ore. Obtaining the platinum group metals by processing waste materials is therefore extremely beneficial for environmental and economic reasons. However, it should be remembered that as a result of the leaching of this type of material, solutions are formed, characterized by high content of accompanying base metals. Nevertheless, the appropriate optimization of the parameters of the extraction and re-extraction process enables the effective separation of precious metals in pure form, even from multi-component systems. Moreover, many corporations have already developed commercial processes for the recovery of the platinum group metals from secondary sources, where liquid extraction is a key technique to obtain these metals quickly and efficiently, while maintaining high purity of the final product.

## Figures and Tables

**Table 1:** The PGMs’ chlorocomplexes.

Ru^3+^	[RuCl_6_]^3−^	Rh^3+^	[RhCl_6_]^3−^	Pd^2+^	[PdCl_4_]^2−^
	[RuCl_5_(H_2_O)]^2−^		[RhCl_5_(H_2_O)]^2−^		
	[RuCl_4_(H_2_O)_2_]^−^			Pd^4+^	[PdCl_6_]^2−^
	[RuCl_3_(H_2_O)_3_]	Ir^3+^	[IrCl_6_]^3−^		
			[IrCl_5_(H_2_O)]^2−^	Pt^2+^	[PtCl_4_]^2−^
Ru^4+^	[RuCl_6_]^2−^				
	[Ru_2_OCl_10_]^4−^	Ir^4+^	[IrCl_4_(H_2_O)_2_]	Pt^4+^	[PtCl_6_]^2−^
	[Ru_2_OCl_8_(H_2_O)_2_]^2−^		[IrCl_6_]^2−^		
Os^4+^	[OsCl_6_]^2−^				

(Source: compiled from [3]).

**Table 2:** The extraction mechanisms of PGMs’ chlorocomplexes.

Solvation	MCl_y_^n−^ + nH^+^ + b[HA] → [H_n_MCl_y_ (HA)_b_]
Ion exchange	MCl_y_^n−^ + n[R_3_NHCl] → [(R_3_NH)_n_(MCl_y_^n−^)] +nCl^−^MCl_y_^n−^ + nH^+^ + n[C][A] → [C_n_MCl_y_] + nHA
Compound formation	PdCl_4_^2−^ +2[L] → [PdCl_2_L_2_] + 2Cl^−^

C—cationic part; A—anionic part; L—ligand; R_3_N—tertiary amine. (Source: compiled from [24,27]).

**Table 3:** Popular extractants for PGM extraction.

Extractant	Starting Solution	Extraction Efficiency	Stripping Solution	Stripping Efficiency	References
Trioctylamine C_24_H_51_N Trade names: Alamine 336; Alamine 300; Alamine 308	Synthetic chloride solution containing Pt and Rh	97% Pt 21% Rh	8 M HNO_3_ (50 °C)	~100% Pt <1%Rh	[42]
	Synthetic chloride solution containing Pt and Pd	>99% Pt >99% Pd	(1) 0.1 M NaSCN (2) 0.1 M TU in 0.5 M HCl	(1) 99.9% Pt (2) 99.5% Pd	[43]
	Pt and Rh salts obtained by leaching of waste materials and dissolved in ethylene glycol	~100% Pt	-	-	[6]
Tributyl phosphate C_12_H_27_O_4_P	Synthetic chloride solution containing Pt, Pd, Mn, Fe, Cr	99.9% Pd	0.5 M TU in 0.1 M HCl	99.8%	[44]
Triisobutylphosphine sulfide C_12_H_27_PS Trade name: Cyanex 471X	Chloride solution obtained after spent catalyst leaching	~100% Pd	0.1 M TU in 5% HCl	46%	[45]
Trioctyl phosphine oxide C_24_H_51_OP Trade name: Cyanex 921	Solution obtained after leaching of mixed spent catalyst samples in aqua regia	>95% Pt	(1) 5.2 mM citric acid (2) 5.5 mM phenanthroline (3) 1.6 mM (NaPO_3_)_6_	(1) 98.1% (2) 63.7% (3) 72.6%	[46]
Mixture of trialkyl phosphine oxides with n-octyl and n-hexyl chains C_42_H_90_O_2_P_2_ Trade name: Cyanex 923	Chloride solution obtained after leaching of glass industry scraps containing a small amount of Fe(III)	85% Pt	(1) 0.1–0.5 M NaSCN (2) 0.01 M HCl	(1) ~100% Pt (2) ~100% Pt	[47]
5,8-diethyl-7-hydroxyldodecane-6-oxime C_16_H_33_NO_2_ Trade name: LIX63	Chloride solution containing Pd, Pt, Ir and Rh	99.9% Pd	(1) 0.5 M TU (2) 0.5 M NaSCN	(1) 99.9% (2) 90.1%	[21]
2-hydroxy-5-nonylacetophenoneoxime C_17_H_27_NO_2_ Trade name: LIX 84I	Synthetic chloride solution containing Pd	>95% Pd	-	-	[31]
Mixture of 5-nonylsalicylaldoxime and 2-hydroxy-5-nonylacetophenone oxime C_16_H_25_NO_2_; C_17_H_27_NO_2_ Trade name: LIX 984	Synthetic chloride solution containing Pd	>97% Pd	-	-	[31]
Mixture of tricaprylylmethylammonium chloride and trioctylmethylammonium chloride C_25_H_54_ClN Trade name: Aliquat 336	Synthetic chloride solution containing Pt, Rh, Al, Mg, Fe	99.97% Pt	0.1–0.5 M TU in 0.1–0.5 M HCl	97–100%	[48]
	Synthetic chloride solution containing Pt, Pd, Mn, Fe, Cr	99.83% Pt	0.5 M TU in 0.5 M HCl	99.9%	[44]
	Solution obtained after spent catalyst leaching in aqua regia	>99% Pt	Na_2_S_2_O_3_	>99.9%	[32]
	Pt and Rh salts obtained by leaching of waste materials and dissolved in ethylene glycol	~100% Pt	1.0 M TU	~100%	[6]
Trihexyl(tetradecyl)phosphonium chloride C_32_H_68_ClP Trade name: Cyphos IL 101	Synthetic chloride solution containing Pt, Pd, Ru and Rh	>95% Pt/Pd ~60% Ru <15% Rh	(1) 0.1 M TU in 0.5 M HCl (2) 1.0 M HNO_3_	(1) >90% Pd (2) >65% Pt	[33]
	Chloride solution obtained after spent catalyst leaching	~100%	1.0 M HNO_3_(used to wash out other base metals, mainly Fe, Pb, Zn and Mg)	Pt 18.6%	[34]
	Synthetic chloride solution containing Pt, Pd, Rh	99.9% Pt 98.1% Pd <2% Rh	(1) 0.02 M TU in 5% HCl (2) 0.1 M NaSCN (3) 0.01 M NH_4_OH	(1) ~100% Pd 10.5% Pt (2) 92.8% Pt <2% Pd (3) 94.6% Pt 81.9% Pd	[35]
	Synthetic chloride solution containing Rh and Pd	99.9–89.7% Pd	-	-	[36]
	Pt and Rh salts obtained by leaching of waste materials and dissolved in ethylene glycol	~100% Pt	1.0 M TU	~100%	[6]
Trihexyl(tetradecyl)phosphonium bromide C_32_H_68_BrP Trade name: Cyphos IL 102	Synthetic chloride solution containing Pd	>80~100% Pd	0.5 M NH_4_OH	84–90%	[49]
Trihexyl(tetradecyl)phosphonium bis(2,4,4-trimethylpentyl)phosphinate C_48_H_102_O_2_P_2_ Trade name: Cyphos IL 104	Synthetic chloride solution containing Pd	52–96% Pd	0.5 M NH_4_OH	~90%	[50]
Trioctyl(dodecyl) phosphonium chloride (P_88812_Cl) C_36_H_74_ClP	Synthetic chloride solution containing Pt, Pd and Rh	Pt, Pd: 99.9% Rh: 10.0–90.0%	(1) 5.0 M HNO_3_ (2) 1.0 M TU (3) 5.0 M HCl	(1) 74.9% Pt (2) 91.2% Pd (3) 73.7% Rh	[23]
	Synthetic chloride solution containing Pd and Rh and contaminants including Al., Mg, Ce, Ba	~100% Pd 80% Rh	(1) 1.0 M TU (2) 5. 0 M HCl	(1) >90% Pd (2) >75% Rh	[51]

TU—thiourea.

## Data Availability

The data presented in this review are based on data collected from the scientific literature and the knowledge and experience of the authors. The sources used in this publication were selected by searching the ScienceDirect, Scopus and Web of Science databases.
